# Dynamics of Population Immunity Due to the Herd Effect in the COVID-19 Pandemic

**DOI:** 10.3390/vaccines8020236

**Published:** 2020-05-19

**Authors:** Vicente Javier Clemente-Suárez, Alberto Hormeño-Holgado, Manuel Jiménez, Juan Camilo Benitez-Agudelo, Eduardo Navarro-Jiménez, Natalia Perez-Palencia, Ronald Maestre-Serrano, Carmen Cecilia Laborde-Cárdenas, Jose Francisco Tornero-Aguilera

**Affiliations:** 1 Faculty of Sports Sciences, Universidad Europea de Madrid, 28670 Madrid, Spain; josefrancisco.tornero@universidadeuropea.es; 2 Grupo de Investigación en Cultura, Educación y Sociedad, Universidad de la Costa, Barranquilla 080002, Colombia; 3 Studies Centre in Applied Combat (CESCA), Toledo 45007, Spain; ajhh1983@gmail.com; 4 Departamento de Didáctica de la Educación Física y Salud, Universidad Internacional de La Rioja, Logroño 26006, Spain; manuel.jimenez@unir.net; 5 Facultad de Ciencias Sociales y Humanas, Universidad de la Costa, Barranquilla 080002, Colombia; jbenitez@cuc.edu.co; 6 Facultad de Ciencias de la Salud, Universidad Simón Bolívar, Barranquilla 080005, Colombia; enavarro27@unisimonbolivar.edu.co (E.N.-J.); rmaestre5@unisimonbolivar.edu.co (R.M.-S.); 7 Nonaffiliated Independent Researcher, Barranquilla 080005, Colombia; nataliaperezpalencia@gmail.com; 8 Vicerrectoría De Investigación e Innovación, Universidad Simón Bolívar, Barranquilla 080005, Colombia; cacelaca6@gmail.com

**Keywords:** SARS-Cov-2, COVID-19, herd immunology, vaccines, pandemic, epidemiology

## Abstract

The novel Coronavirus 2 Severe Acute Respiratory Syndrome (SARS-Cov-2) has led to the Coronavirus Disease 2019 (COVID-19) pandemic, which has surprised health authorities around the world, quickly producing a global health crisis. Different actions to cope with this situation are being developed, including confinement, different treatments to improve symptoms, and the creation of the first vaccines. In epidemiology, herd immunity is presented as an area that could also solve this new global threat. In this review, we present the basis of herd immunology, the dynamics of infection transmission that induces specific immunity, and how the application of immunoepidemiology and herd immunology could be used to control the actual COVID-19 pandemic, along with a discussion of its effectiveness, limitations, and applications.

## 1. Background and Basis of Herd Immunity

The term herd immunity was first used in 1923 by Topley and Wilson [1]. It subsequently served as the basis for vaccines and their applications, vaccination program cost analyses, and the eradication of diseases such as smallpox and infectious diseases such as polio and diphtheria [2,3,4]. The concept of herd immunity is used to describe the immune ratio between individuals in a population, the threshold of immune individuals that will lead to a decrease in disease incidence, and the pattern of immunity that will protect a population against a new infection [3]. Herd immunity depends on factors such as population immunity and the dynamics of the transmission of etiological agents [4,5]. Different studies have analyzed the effectiveness of vaccination programs to achieve herd immunity in, and thus protect the unvaccinated or immunocompromised against different diseases [6]. The most representative examples are the vaccines for cholera, Hepatitis A, Hepatitis B, Human papillomavirus, Hemophilus influenza, Meningococcal, Influenza, Pneumococcal, Polio, Whooping cough, Measles, Chickenpox, Rotavirus, and Yellow fever [7,8,9,10,11,12,13].

Herd immunity has also been studied in bacterial communities, where it is important because it allows the stable coexistence between bacteria and their phages, as well as the maintenance of polymorphisms in bacterial immunity [14]. The impact of herd immunity on the efficiency of disease transmission was recently observed in America with the Chikungunya and Zika viruses, which caused an epidemic in a fully susceptible population. However, after the introduction of these arboviruses, herd immunity limited their transmission [15]. Similarly, the United Kingdom recently implemented a strategy for the SARS-CoV-2 pandemic that caused great controversy, allowing the virus to spread in the population before ordering social isolation to increase herd immunity [16].

## 2. Herd, Population, Collective, or Group Immunity

Interpreting the scientific concept of collective/group immunity as the resistance that a certain community possesses against an infection, Fox pointed out four conditions under which such immunity can occur [17]. Initially, the infectious pathogen must be found and restricted to a single host. For severe acute respiratory syndrome coronavirus 2 (SARS-Cov-2), the primary transmission was zoonotic, with its natural reservoir being the bat. However, the intermediate host through which it was transmitted to humans is uncertain (a ferret is hypothesized). Transmission is believed to have occurred due to direct contact with the infected animals or through their secretions. Viral RNA has also been found in dogs and cats living with coronavirus disease 2019 (COVID-19) positive humans. However, it has not been shown that these animals can transmit the virus to people [18].

As the second condition, the transmission must occur primarily through direct contact. To date, it has been established that the transmission of SARS-Cov-2 occurs by direct, person-to-person contact (coughing, sneezing, and inhalation of droplets) and contact transmission (contact with the oral, nasal, and ocular membranes) [19]. Likewise, viral RNA has been identified in the feces of COVID-19 positive humans with gastrointestinal manifestations, which could be another route of transmission of the virus [20]. Previous studies of pregnant women did not find evidence of vertical transmission or fetal and perinatal complications [21].

Third, the infection must induce solid, long-lasting immunity. Given the limited information on the immune response induced by SARS-Cov-2 in humans, it has not been possible to establish the mechanism by which the immune system generates a long-term response that could combat the disease and prevent reinfection. Experimental animal studies have tested the reactions of the immune system in Rhesus monkeys, finding the generation of neutralizing antibodies, thereby suggesting that monkeys with the disease would not be able to re-infect themselves with the same virus strain and increasing expectations and hopes for a future vaccine. [21]

Finally, collective or group immunity is maximized if the population possesses a random mixing pattern. In this case, everyone is susceptible to contracting the SARS-Cov-2 virus, with or without COVID-19 symptomatology. However, this pattern of random mixing will depend on the measures implemented by each government worldwide (e.g., quarantine, isolation, and social distancing, and reinforced preventive measures for at-risk groups such as pregnant women, the elderly, and children [22]. Theoretically, it is possible to achieve collective immunity under the aforementioned assumptions. However, at present, our unawareness of the adaptive immune response and, in the absence of a vaccine, the ethical limitations to achieve such immunity make this solution too unethical to consider [23].

## 3. Person-to-Person Transmission Dynamics Inducing Specific Immunity

**Figure 1:**
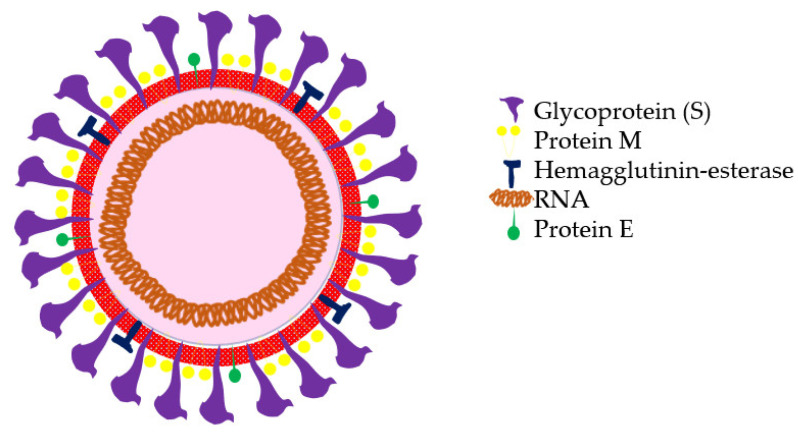
Molecular structure of severe acute respiratory syndrome coronavirus 2 (SARS-CoV-2).

Coronaviruses are members of the Orthocoronavirinae subfamily within the Coronaviridae family (order Nidovirales). This subfamily comprises four genera—Alphacoronavirus, Betacoronavirus, Gammacoronavirus, and Deltacoronavirus, according to their genetic structures. Alphacoronaviruses and betacoronaviruses infect only mammals and are normally responsible for respiratory infections in humans and gastroenteritis in animals. Structurally, coronaviruses are spherical viruses 100–160 nm in diameter, with a lipid bilayer envelope containing positively polarized single-stranded RNA (ssRNA) between 26 and 32 kilobases in length. The SARS-CoV-2 virus genome encodes four structural proteins—protein S (spike protein), protein E (envelope), protein M (membrane), and protein N (nucleocapsid) (Figure 1).

Protein N is located inside the virion associated with viral RNA, and the other three proteins are associated with the viral envelope. Protein S forms structures that protrude from the envelope of the virus. Protein S contains the receptor-binding domain of the cells it infects and is, therefore, the virus’s tropism-determining protein. In addition, this is the protein that provides the fusion activity between the viral membrane and the cellular membrane, which allows the viral genome to be released inside the cell that it is going to infect.

In the pandemic caused by SARS-CoV-2, the entire population worldwide is susceptible to acquiring the COVID-19 disease, as there is no herd immunization to the virus. Three possible routes of transmission have been described: (i) droplet transmission, (ii) direct contact transmission, and (iii) aerosol transmission. However, recently the digestive system has also been marked as a potential transmission route based on abdominal manifestations and symptoms of diarrhea caused by the virus, as well as the viral RNA found in feces [24].

Several factors may influence the virus’s transmission given the infectivity of the host, such as behavioral factors (e.g., isolation, hand washing, and self-care behaviors), which are necessary to attenuate the probability of infection and contagion. It has been reported that public health interventions that block more than 60% of transmissions are effective to control the containment of the COVID-19 outbreak [25].

Likewise, different studies have found that the at-risk populations more susceptible to the disease are the elderly, males, pathological patients with the presence of hypertension, those with poor or depressed immune system functions, and users of long-term immunosuppressive agents [26]. Likewise, for the infected population, the possibility of being asymptomatic could range, depending on the scenario and population, from 5% to 80% [27], with a possibility of transmission to healthy hosts for up to 14 days, which should translate into more careful suppression measures by governments [28].

Regarding the epidemiological characteristics of the disease, different COVID-19 studies have estimated a basic reproduction range (R0) of 2.2 to 4.71 [29]. The R0 may vary according to the governmental public health measures taken during the different phases of the epidemic [30], as well as the different methods used to calculate the R0 (e.g., the epidemic growth rate of the curve and the serial interval) and the validity of the underlying assumptions, different scenarios, and levels of zoonotic exposure [31]. Therefore, different methods, measures, contexts, and scenarios can lead to differences in the epidemiological data [32]. The present data may lead to biased estimations. However, epidemiologists affirm that as more data become available, these biases may decrease, with the actual R0 expected to be 2.2–4.71, according to the World Health Organization (WHO) estimations (R0: 1.95 IC: 1.4–2.5) [33].

Another important parameter for understanding population immunity is the effective reproductive number (Rt), which refers to the average of secondary cases generated by a single index case during an infectious period in a partially immune population, which, unlike R0, does not fully assume a susceptible population and will vary depending on the current immunity of the population [34]. For Covid-19, the estimate of the Rt has varied by country due to the intrinsic and extrinsic factors of each population. For South Korea, an Rt of 1.5 was calculated (95% CI: 1.4—1.6) [35]; for the United States, 3.29 (95% CI: 3.15—3.43); for Italy, 2.44 (95% CI: 2.41—2.47); and for Brazil, 3.26 (95% CI: 2.99—3.55) [16], meaning that the minimum proportion of the total population needed to confer herd immunity will vary according to the estimated Rt for each country.

The average time of the SARS-CoV-2 incubation process in humans has been debated by authors. The first studies suggested seven days [36], while the most recent studies have made estimations of less than three days, with COVID-19 symptoms such as pneumonia presenting after the fourth day of incubation [2,3,4,5,6,7,37]. Currently, there are no vaccines to face the pandemic. It is hoped that as more data on the disease become available, specific immunity mechanisms will be able to control the infection enough to prevent the development of the disease. Without a vaccine, we expect to achieve collective immunity when approximately 70% of the population has been infected, which would make it easier for at-risk populations to protect themselves from infection to a certain extent—not due to specific immunity but because the probability of exposure to the infectious pathogen is decreased.

## 4. Applied Immunoepidemiology for Epidemic Control Strategy Development

Since the appearance of SARS-Cov-2 in the city of Wuhan (Hubei, China) in December 2019, governments around the world have taken unprecedented actions to contain it [38]. Countries are implementing different community, economic, and public health control measures to flatten the epidemic curve and avoid overloading and possibly collapsing their health systems. The control measures that countries established since the WHO characterized COVID-19 as a pandemic [39] depend on each area’s culture, health systems, the magnitude of the epidemic, and the control that the governments exercise over their citizens; each response is also influenced by weaknesses in epidemiological surveillance systems that must generate real-time information to allow decisions to be made to mitigate the impact of the epidemic outbreak [40].

An example of an approach that combines the use of traditional epidemiological surveillance techniques with new clinical data entry codes specific for COVID-19 is the method developed by the Royal College of General Practitioners (RCGP), Research and Surveillance Center (RSC), and the Public Health England (PHE). This system uses five components of epidemiological surveillance: (i) the collation of data from the clinical records of patients with suspected exposure to COVID-19 attended to by one of the 500 doctors who are part of the RCGP RSC, (ii) the referral of suspected patients to the national emergency system from the primary care provided by RCGP RSC physicians, (iii) the collection of serological samples from all age groups, (iv) the collection of samples from possible patients with clinical signs of pneumonia, and (v) the collection of clinical data from recovered individuals. This allows officials to test the effectiveness of COVID-19 measures [41].

Most countries follow a traditional approach to the epidemiological surveillance of infectious diseases based on identification, tracking, and testing individuals at high risk with periodic reports of infection (as well as the recovered and dead) by country [42]. This allows the treatment and isolation of the sick and the quarantine of individuals with suspected cases. However, it does not allow us to predict cases or offer an objective overview of the situation to inform decision-making for the distribution of medical resources and the implementation of community control measures [40].

Risk-based surveillance among high-risk individuals improves the ability to detect the few expected new cases as early as possible by targeting those who are more likely to be infected than others. In a very low prevalence, or early epidemic scenario, risk-based surveillance is cost-effective and is more likely to encounter cases than survey-based random surveillance because resources are directed at high-risk subpopulations [40]. The utility of risk-based epidemiological surveillance data is reduced once the infection is established because the identified cases are not representative of the infected individuals in the population.

Social isolation and surveillance measures must be followed in all countries based on the indications of the WHO. Health authorities should promote the universal monitoring of temperature, masking, and handwashing based on the WHO recommendations [42]. To prevent imported cases from spreading locally, border control measures should include the temperature detection of all passengers on flights and an obligation of social isolation for at least 14 days, just as Singapore, Korea, and Japan did since the beginning of the epidemic with travelers from mainland China and Japan [39]. In Singapore, the response to the novel viral infection was early—temperature measurements for travelers from Wuhan began on the 3rd of January, 2020. In Japan, temperature measurement measures for travelers from China began on 7 January 2020 and in South Korea on 1 April 2020 [42].

The provision of personal protective equipment, such as masks, for medical institutions that perform PCR tests and admit patients for hospitalization, should be prioritized. Secure processing and reporting must be done promptly via PCR tests [43,44]. Rapid/point-of-care diagnostics and serologic assays must be quickly developed and evaluated. The human rights of health personnel must be protected both in hospitals and in daily life, where they can suffer aggression from the community [45].

It is recommended to monitor in real-time the epidemic impact of the disease on health systems to distribute resources efficiently and to monitor viral changes to generate useful information for the development of vaccines and pharmacological treatments, identify possible viral mutations, and determine markers of serious infection [46]. In this way, the WHO is monitoring the SARS-CoV-2 spread very closely via a global surveillance system. Apart from WHO surveillance, the governments of various countries, as well as their health ministries, home ministries, aviation ministries, and non-government organizations, are working together to track the latest developments in COVID-19 cases [47]. Among them are the European Center for Disease Control and Prevention (ECDC), the Johns Hopkins Center for Systems Science and Engineering (CSSE), the State Council Information Office in Beijing, and Center for Disease Control and Prevention (CDC) [48].

Other strategies may involve the use of online medical platforms for routine cases and follow-ups with patients, if possible. These measures also include moving to virtual teaching for college and university students and canceling mid-year school vacations. Other measures include the following: focusing on the reduction of clusters of cases, thoroughly controlling the epidemic, and striking a balance between epidemic prevention and control and sustainable economic and social development and using big data, artificial intelligence (AI), and geo-positioning and tracking applications to strengthen contact tracking and the management of priority populations. However, the use of user positioning technology through connection to Wi-Fi networks may have ethical and legal implications (i.e., a violation of privacy) [49]. Finally, the creation and implementation of bot-based applications to keep the population informed about key aspects of the COVID-19 is a possible approach.

The following is a summary of epidemiological surveillance measures adopted by different continents (e.g., Asia, Europe, and America (Table 1)), including the application of molecular biology and rapid tests based on antigens and antibodies for COVID-19 detection, social isolation, and the use of technology to keep the population informed and controlled at all times.

## 5. Herd Immunity Applications in the Current COVID-19 Pandemic

The question remains: What will be the impact of herd, collective, or group immunity as a mitigation measure on the dynamics and progression of the viral COVID-19 pandemic?

**Figure 2:**
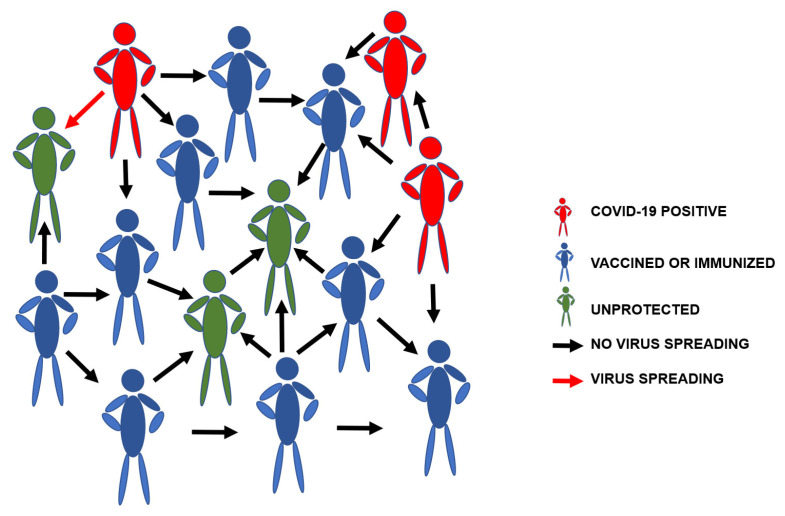
Herd immunity for the spread of severe acute respiratory syndrome coronavirus 2 (SARS-CoV-2).

According to different models applied to infectious diseases, herd immunity is mathematically related to the propagation and infection tendencies of the virus [50,51], which are the result of the relationship established over a certain period of time between the number of healthy subjects and those susceptible to infection, the infected subjects that can no longer contribute to the transmission of the virus, infected subjects, and the natural or vaccine immune subjects (or resolved cases by death) in a population, mediated by the infectiousness of the microorganism, the incubation period, the transmissibility period, the contagion capacity between people, the dynamics of contact between the population, and the duration of the disease [52]. A spread model of herd immunity for SARS-CoV-2 is shown in Figure 2.

Based on this knowledge, the mathematical expressions for the basic reproduction number R0 and the effective reproduction number (Re) can be derived. These numbers allow us to compare the speed at which the disease grows to the capacity of recovery against the number of susceptible cases to determine the Critical Immune Fraction (which is necessary to reach in order to develop immunity), as well as the time that it would take to develop, avoid, or control the spread of the disease [53].

Related to the concept of infection reproducibility, it is also mathematically possible to quantify a virus’s transmissibility (i.e., to define whether the disease can succeed in spreading or not), as well as its endemicity (i.e., the fraction of susceptible cases necessary for it to become endemic (stable)). The higher the R0, the higher the virulence, and the higher the level of immunization or population immunity needed to ensure control [54].

**Figure 3:**
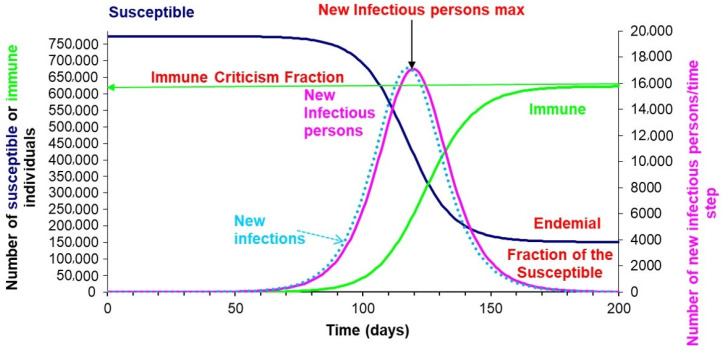
Susceptible, exposed, infected, and resistant (SEIR) model simulation of the coronavirus disease 2019 (COVID-19) propagation dynamics in a standard population with random circulation, with 770,000 susceptible, one infectious, and zero recovered at time 0. Key input parameters in the model: Average pre-infectious period (days) = 2, Average duration of infectiousness (days) = 5.6, and R0 = 2 [44].

Based on the dynamics of the established relationships, in the absence of a vaccine, protective measures, or treatment alternatives, it is logical to assume that at the beginning of the spread of SARS-CoV-2 in a standard population with random contacts and zero people recovered or immune, there would be a rapid growth of infected cases, reaching the maximum number of cases per unit of time at the point on the Cartesian plane where the line of decreasing susceptibility crosses that of those recovered or resolved in growth and reaching a level of endemicity (stability) when the critical immunological fraction or the population immunity necessary for probable infection among the susceptible cases trends toward zero [55] (Figure 3).

The simulated susceptible, exposed, infected, and resistant (SEIR) model represented in Figure 3 assumes that to prevent and control the COVID 19 pandemic, the number of individuals susceptible to infection must be reduced until population immunity is achieved (in the aforementioned simulation, this number is approximately 74%). Theoretically, this would mean that once the fraction of people with naturally acquired immunity in the standard population exceeds 0.74, the number of newly infected people would start to decrease. Such propagation assumes a standard population with random contacts and uniform distribution in different population groups according to their state (SEIR), which, despite being unlikely, allows us to determine the critical immunological fraction necessary to achieve population immunity in the absence of vaccines [33].

However, the characteristics of the present pandemic at the moment provides some major challenges to public health, institutions, and governments, related not only to the dynamics of the propagation and contagion but also to the great impact it has had on the population (morbidity and lethality) due to the exponential rise in the number of those infected, whose volume of required health services exceeds the capacity of their country’s health systems, as seen in France, Italy, Spain, the United Kingdom, the United States, and Ecuador, among others [56].

Taking into account the aforementioned considerations, it is necessary to design and implement innovative and integrative interventions that, without neglecting studies on the immune dynamics and application of strategies to generate population immunity, are accompanied by interventions that reduce the R0 (barrier measures, quarantine, physical distancing, and effective treatments), allowing us to achieve intelligent suppression–mitigation and subsequent control of the COVID-19 disease [57].

## 6. Herd Immunity Limitations in the Current COVID-19 Pandemic

The COVID-19 pandemic is now a major global health issue, representing the most serious respiratory virus since the 1918 H1N1 influenza pandemic, whose lethality ratios are comparable [58]. Although our knowledge and understanding of how infectious diseases can affect populations and spread have increased since 1918, it seems that we are not still prepared to properly confront a worldwide pandemic. Governments are quickly responding with different counter measurements, as epidemiologists estimate 7.0 billion infections and 40 million deaths globally for the year 2020 if no-interventions are made [59]. To date (5 May 2020), the ongoing SARS-Cov-2 has 3.646.834 active infected cases, with a total of 1.200.573 recovered patients and 252.442 deaths. Thus, there are two possible alternatives to stop the COVID-19 pandemic—suppression and mitigation.

(1) Suppression measures are those based on attenuating reproduction/infection cases, forcing the R0 of the virus below R1 with the use of non-pharmaceutical interventions (NPIs) in a continuous or intermittent way for as long as the virus is circulating or until a vaccine is available, which, according to recent data, is likely at least 12–18 months away [60]. These types of measures can only delay the pandemic outbreak, not prevent it since there is no herd immunity acquired by the population. A clear example of this method is an extraordinary restriction on free movement and assembly, remaining vigilant and being willing to completely quarantine people combined with aggressive and extensive testing, including a testing infrastructure to test even those who are not sick. Suppression strategies will allow the scientific community and governments to buy time until a vaccine or an effective treatment is ready and becomes widely available worldwide. However, if controls are eased or restricted, the pandemic is likely to re-emerge as no herd immunity has been acquired.

(2) Mitigations measures, on the contrary, do not aim to interrupt the transmission completely but instead to progressively attenuate the impact of the epidemic, thus reducing the R0 of the COVID-19 virus, estimated at 2–3 [61] but not below 1. These measures will allow the epidemic to proceed at a controlled rate, keeping demand on the healthcare system under capacity, and affording herd immunity [62]. Examples of this strategy include the UK and the US, with their “laissez-faire attitudes”.

### 6.1. Laissez-Faire Attitudes or Natural Herd Immunity

This type of mitigation measure is a classical concept that can be found in epidemiological books and has been successfully applied in cases such as the 1918 H1N1 influenza pandemic, as there was no vaccine for the novel infectious disease [63]. Herd immunity may be achieved when (i) the population is exposed to the disease and builds up a natural immune response and defense mechanism to the virus or (ii) when the population is vaccinated against the disease to achieve immunity. Thus, by vaccinating certain groups of the population, the spread and R ratios of the virus will go down.

In the absence of a vaccine, building herd immunity against SARS-Cov-2 through natural infection is theoretically possible. However, there is no ethical path to reach this goal, as the social consequences of natural exposure may be devastating. Indeed, current mathematical and epidemiological analyses suggest that herd immunity is not the answer to stop the novel coronavirus; exposure to the virus should be avoided until either a vaccine or effective pharmacological treatments are available. For this reason, Pharmacological Interventions (PIs), such as the use of Hydroxychloroquine, Azithromycin, interferon-α, Lopinavir, Ritonavir, Ribavirin, Chloroquine phosphate, and Arbidol, have been suggested to be potentially effective in combating COVID-19 once we know the virus’s genetic sequence and mechanism of infection. However, the efficacy and safety of these candidate drugs in the treatment of COVID-19 need to be confirmed in further preclinical and clinical trials, despite the in vitro studies and non-clinical trials already published [64,65,66].

### 6.2. Possible Consequences of Obtaining Herd Immunity by Natural Pathways

There are two epidemiological concepts that need to be clarified: (1) the overall case fatality rate (CFR), which is the proportion of deaths attributed to a certain disease among all individuals diagnosed with the disease over a specific period of time, and (2) the infection fatality rate (IFR), which is defined as the proportion of deaths caused by a certain disease among individuals. The combination of both can project the expected number of deaths as a consequence of meeting the herd immunity threshold [67]. Scholars have determined that with a uniform herd immunity threshold of 67% (R0 = 3) and an IFR of 0.6%, the absolute number of expected deaths worldwide would exceed 30 million citizens [34]. In this context, several health care systems may collapse, as in Italy, during its early stages [68]. In countries such as the US, the “laissez-faire” (no action taken) approach of developing herd immunity via natural pathways could lead to 2.4–21 million citizens at the hospital, with a fatality rate of 3.5%, according to the CDC [69]. This would also lead to an increase in all-cause mortality due to a lack of resources for patients that need primary/urgent attention by causes unrelated to SARS-Cov-2.

### 6.3. Epidemiological Limitations

Many elements may affect the R0, such as population density (virus transmission centers are found in large urban nuclei, such as London, Madrid, and New York, compared to the rest of the country’s national territories), cultural behaviors, and the average population age [70,71]. In addition, the presence of previous chronic diseases and pathologies is an important epidemiological limitation. The risk of death is increased with comorbid cerebrovascular diseases (22%), hypertension (23.7%), diabetes (22%), coronary diseases (5.8%), or a depressed immune system. Age and sex are another limiting factor, since the COVID-19 fatality rate by age ranges from 0.2% (10–19 years) to 14.8% (80+ years) and by sex (4.7% for males) and (2.8% for females) [72,73,74].

### 6.4. Immunological Limitations

Since this is a novel disease, little is known about it. One of the most important questions when discussing herd immunity is the following: Can a patient get reinfected? This question remains unclear since after the outbreak and control of the epidemic in China, recovered patients have again tested positive [60]. However, viral RNA can last for a long period of time, even after the actual virus has been stopped, thus allowing studies to detect residual viral RNA without a live virus. Recent studies in rhesus macaques have shown that infection with SARS-CoV-2 could protect from subsequent exposure, thereby avoiding reinfection and offering safe re-exposure to the viral environment [75]. However, even if reinfection is possible after immunity wanes, the enduring memory cells of the immune system will likely (in a healthy patient) facilitate immune control of the virus, limiting its spread and effects and reducing the clinical severity of subsequent infections.

Considering the current literature, we conclude that herd inhumanity via natural pathways should not be the ultimate goal since millions of infected would perish from the virus. The only viable and ethical pathway is by using suppression measures until a vaccine is available worldwide. The successful mitigation policies of China and South Korea were possible due to extremely intensive measures, including mandatory and strictly enforced quarantine, large amounts of resources devoted to contact tracing, the electronic surveillance of citizens’ movements, etc. These cases of success should be studied as an example, with the possibility to implement them in occidental cultures.

## 7. Herd Immunity Effectiveness

According to the WHO, herd immunity only works for contagious diseases [76]. This means that it cannot work for diseases such as tetanus, where anyone who is not vaccinated could easily be infected if exposed to the bacteria, even if everyone else around them is vaccinated and protected. For contagious diseases, however, we must not only protect the immunized but also reduce disease among unimmunized individuals through herd protection. Herd protection occurs when a sufficient proportion of the group is immune [77]. The coverage rate necessary to stop transmission depends on the basic reproduction number, defined as the average number of transmissions expected from a single primary case introduced into a susceptible population. The prevalence of transmission will decline if the fraction of susceptible hosts is low. When the proportion of susceptible hosts falls below the threshold needed for transmission, this is known as the immunity threshold [78].

Due to herd protection, some diseases can be eliminated without 100% immunization coverage. Haemophilus influenzae type b vaccine coverage of less than 70% in the Gambia was enough to eliminate Hib disease, with similar findings in Navajo populations [79,80]. In the US measles outbreak among preschool-age children, the attack rate decreased faster than the coverage increase [81].

For SARS-CoV-2, herd immunity, while proven effective, might not mitigate the virus. The results depend on the timing and efficacy of the control measures and the ability to bring rapidly growing outbreaks under immediate control when needed [82]. Vaccines for this virus are not yet available, but current pharmacological interventions such as azithromycin and hydroxychloroquine (among others) have been suggested to be potentially effective. However, their efficacy and safety must be confirmed in further studies for this novel disease [83,84,85]. The main question, then, is if humans can become reinfected. Enhancing herd immunity to control the COVID-19 epidemic is problematic. Given that the case fatality rate (CFR) of COVID-19 can range between 0.25% and 3.0% of a country’s population, the estimated number of people who could potentially die from COVID-19 for the population to reach a minimum (‘critical’) level of population immunity (P crit herd immunity level) may be difficult to accept [86]. Currently, the best way to minimize the loss of lives and the occurrence of severe cases requiring intensive care is to shelter vulnerable groups of individuals and slow the spread of the virus [87].

## 8. Herd Immunity and Vaccines

The new SARS-Cov-2 genome has a 79% similarity with the previous SARS-Cov strain that infected part of the Asiatic Southeast population and caused thousands of pneumonia cases [88]. Since the first infections attributed to SARS-Cov-2 in December 2019, the spread has reached global pandemic levels [36]. To date, close to four million people have been infected in 190 countries, and nearly 270,000 have died as a result of the respiratory complications and inflammatory processes caused by the new coronavirus. Hundreds of scientific articles were published in the first two months after the onset of the virus, showcasing the importance of SARS-Cov-2 to the scientific community. Vaccine development could help stop the spread of the virus, especially among the most vulnerable populations. This goal has become the strategy most intensively pursued by international laboratories [89,90].

Vaccines have traditionally been considered a form of a preventive intervention for direct and indirect protection (i.e., herd immunity) in a target population. Before voluntary vaccination campaigns, infection indicators were lower in rural areas and among those of low socioeconomic status. This natural immunity to certain viral diseases reached only 12% of people [91]. Progressive vaccination campaigns and improvements in comprehensive sanitation in urban settings have helped to improve herd immunity [92]. On a large scale, the indirect effects of vaccines have been observed and measured through increasingly sensitive and precise parameters, thereby achieving scientific consensus about their contributions to public health and the high safety of inoculation [93]. It is important to note that immunization is also modulated by vaccine type, the individual organism response, prevention program adherence, and the age of administration [94].

**Figure 4:**
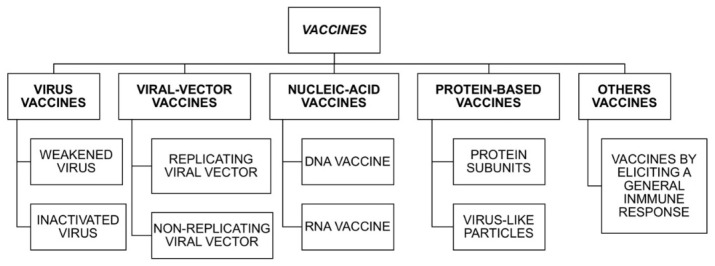
Severe acute respiratory syndrome coronavirus 2 (SARS-Cov-2) vaccine variety, adapted from Callaway (2020), pp. 576–577.

Vaccines train the body to recognize and fight a specific pathogen. The viral spread is restricted when the vaccination rate or the prevalence of a high percentage of positive serological individuals in the population seriously limit the transmission of the infectious agent from one human to another. Establishing a “critical” population immunity percentage to curb the expansion of COVID-19 is, with current scientific knowledge, purely speculative. Hence, if the R0 of SARS-Cov-2 fluctuates between 2 or 3, herd immunization would require infection of about 50% to 70% of the world’s population to keep the disease under control (or close to 80% if the R0 is higher). In addition, the mass vaccination of billions of people could be one of the most important global challenges of the 21st century [16]. Currently, 100 vaccines are being developed to combat SARS-CoV-2, with funding mainly coming from private pharmaceutical companies (70%) and, to a lesser extent, public capital (especially with contributions from university research centers). Figure 4 shows the different vaccine options currently identified [95]. Some research teams are using inactivated or weakened viruses, but most studies on vaccine candidates focus on vaccines based on viral proteins, nucleic acids, or synthetics.

Today, the progressive increase in aging, especially in industrialized countries, has produced new public health challenges. Most people over 65 have not completed vaccination programs, or the substances used to vaccinate them were not as effective and safe as those in current vaccines. In addition, many old people have comorbidities that lower their immune systems or a lack of physical energy to deal with viral infections [96]. The novel SARS-Cov-2 is especially dangerous in people of advanced age or with comorbidities, such as diabetes, coronary problems, cancer, or a depressed immunity system. Other environmental factors, such as diet quality, sedentary lifestyle, and medical treatments, may limit the immune responses of most vulnerable people before future vaccine administration against COVID-19 (e.g., immunosenescence). Consequently, herd immunity may be the most important “short term” strategy to protect this portion of the population [97]. Until a safe vaccine is developed, research on specific new treatments (or an effective combination of existing treatments), together with action plans to contain the spread of the virus, seem to be the only alternatives for protecting at-risk populations [98].

Computer projects have worked on SARS-Cov-2 genome sequencing in collaboration with laboratories and researchers to search for a synthetic vaccine [99]. Different types of vaccines are under development in the United States, Europe, and China [37]. The first strategy could be directed at developing an initial vaccine that stops the spread immediately, while at the same time searching for the best prospective candidate to be administered with higher safety and effectiveness—i.e., vaccines that not only stop the spread but also eliminate the presence of the virus in the nasal and oropharyngeal mucosa and offer long-term permanent immunization [100]. It seems likely that this pandemic will only stop when herd immunization, either by infection and recovery or by creating an effective vaccine, completes the process of viral spread. Several laboratories are working against the clock to achieve direct immunity against COVID-19 in the shortest period of time [97].

Discovering an effective vaccine will not be without certain challenges to overcome, such as its secondary effects, price and ease of accessibility, limited secondary effects to vulnerable people, long-term immune response, and the willingness of the population to be vaccinated voluntarily [77]. There are certain limits to persuading people to engage in mass vaccination [101], even when direct immunization could safely extend indirect immunity to the most vulnerable populations [102,103,104]. Theoretical models suggest that the probability of real, immediate infection increases the population’s commitment to voluntarily submit to vaccination campaigns [105]. However, public communication plans on the importance of herd immunity and easily accessible vaccination campaigns will be necessary to increase adherence to prevention programs in the fight against the COVID-19 disease [58].

## 9. Conclusions

Among humans, novel SARS-Cov-2 transmission occurs by direct person-to-person contact (coughing, sneezing, and inhalation of droplets) and contact transmission (contact with the oral, nasal, and ocular membranes). Herd immunity has long been used for the eradication of many diseases and provides the basis for vaccines and their applications. With the emergence of the novel COVID-19 pandemic, some strategies have included the implementation of herd immunity but caused great controversy.

Effective herd immunity for the COVID-19 pandemic needs to consider some specific conditions. The infectious pathogen has been found, but the intermediate host is still uncertain. Also, there should be long-lasting immunity, which has not yet been possible to establish for SARS-Cov-2. Random mixing of the population would maximize this group immunity but will depend on the measures implemented by each country. It is necessary to design and implement innovative interventions to reduce the R0 to achieve intelligent suppression–mitigation and subsequent control of COVID 19. Without a vaccine in sight, group immunity is expected to be obtained when approximately 70% of the population has been infected. Given the fatality rate, the estimated number of people who could potentially die from COVID-19 may be difficult to accept.

Different types of vaccines are under development worldwide in order to curb the advance of the virus, with the best candidates being the safest and most effective. The COVID-19 pandemic seems likely to come to an end only when an effective vaccine is created and applied, and herd immunization is acquired. Until a vaccine is available, epidemic suppression through extremely intensive measures appears to be the only viable and safe strategy, such as enforcing quarantine and the application of many contact control resources to slow the spread of the virus.

## Figures and Tables

**Table 1:** Summary of epidemiological surveillance measures, use of molecular biology and rapid tests based on antigens and antibodies for coronavirus disease 2019 (COVID-19) detection, social isolation, and the application of technology to keep the population informed and controlled, as adopted by different countries.

Country	Rapid Tests Based on Antigens and Antibodies	RT-PCR Screening Tests	Citizen Information Measures	Geo-Position and Tracking Applications	Quarantine Measures	Use of Masks and Other Safety Measures
Canada	Yes. To date (12 April, 2020), Health Canada has approved this measure (shipping only in Canada, not worldwide). Several biomedical private entities received approval for the production of rapid kits.	Government has a guide for public and private institutions. Performed with a rapid test.	Large governmental web page with answer and questions (FAQ). COVID-CANADA App provides a central resource for accessing personalized, trusted, and evidence-based information.	Authorities are still discussing this. No official information available.	16th of March, borders were quarantined Since the 7th of April, the population has been under lockdown	Suggested mask implementation. Thermal scanning. Plexiglass barriers to ensure social distancing. GPS–Bluetooth technology to determine whether citizens have had contact with a COVID-19 positive subject.
China	Yes. Rapid diagnoses and rapid/point-of-care serological tests, especially in Wuhan province, the focus of the onset of COVID-19.	The results of PCR tests are returned the same day in mainland China and performed on even suspected patients.	No information was obtained.	Yes. Using 4g and 5g connections.	Border health quarantine; infectious diseases on 20 January 2020 in Wuhan and other key areas of Hubei.	Strict entry control in buildings (a green health QR code used to grant access) connected to a governmental database that is informed on the health status and movements of the citizens. Mandatory masks. Thermal scanning. Strict disinfection protocols that are shared on social media to engender feelings of security and comfort among the population.
Colombia	Yes. With limitations in the ability to obtain rapid detection kits.	Yes. With the participation of University laboratories and state epidemiological surveillance. There are problems with the analysis of samples and the reporting of sample results.	Yes. Limited due to the low diffusion of the government. The use of a WHO WhatsApp bot that delivers real-time information to phones.	No.	Yes. From 24 March till 27 April.	Compulsory use of masks on public transport systems and in public areas, such as stores, outdoor marketplaces, and banks.
European Union	Yes. Antigen and antibody-based rapid tests are widely used in the countries of the European Union and the United Kingdom. Their application is recommended to support laboratories that perform RT-PCR tests. The use of commercial tests with a "Conformité Européenne" (CE) seal is recommended. Tests based on direct SARS-CoV-2 antigen detection and indirect antibody detection tests are used.	RT-PCR is the current test methodology applied in EU member states. The ECDC recommends nucleic acid amplification-based tests, such as RT-PCR, as diagnostic tests.	Yes. The use of the WHO WhatsApp bot delivers real-time information to phones.	Europe’s telecom companies are sharing location data with health authorities in Italy, Germany, and Austria to check whether people are remaining at home. The data are aggregated and anonymous, mapping concentrations rather than individuals to respect Europe’s privacy laws.	The authorities of the European Union recommend home isolation for people who have mild symptomatology and quarantine for people who have been potentially exposed.	In the European continent, Bulgaria, Georgia, Luxembourg, Poland, the Czech Republic, and Ukraine have mandatory mask-wearing. Masks are only necessary in enclosed public places, public transport, and shops in Austria, Lithuania, Romania, and Slovenia. From 11 May in France, masks will be mandatory on public transport and recommended for shopping and small areas. In Germany, Spain, and Italy, donning a face mask is also compulsory, including public transport and shops.
Russia	No information available.	Yes, since 27 March, AmpliTest SARS-Cov-2 is used to test for the virus in just 2 h and 30 min.	No information available.	Yes. Only patients who have tested positive and have been hospitalized.	18 March: restrictions on the entry of foreigners.	No information available.
Japan	Yes, but rapid tests are applied to a lesser extent than in other countries. Confirmatory tests of PCR RT are preferred.	86,800 PCR tests have been conducted through to 17 April. Private laboratories can be authorized to perform PCR tests.	Yes. The use of the WHO WhatsApp bot delivers real-time information to phones.	No data available.	Maintained social and economic functions. No cities blockaded as in other countries; only partial quarantine.	Among other countries such as Hong Kong, South Korea, Thailand, and Taiwan with similar cultures, the broad assumption is that anyone could carry the virus; thus, masks are encouraged to be worn at all times and in all places.
United Kingdom	Yes. Since 15 March, rapid tests are available for use in community pharmacies or at home. Two million tests have been bought from China as of the 1st April.	National Health Service (NHS) is providing guidance and standard operational procedures for public and private institutions.	Yes. The use of the WHO WhatsApp bot delivers real-time information to phones.	Yes. A governmental App is used to track the progression of medical conditions by citizens self-reporting their symptoms No information on geo-tracking.	Since 23 March.	The use of a mask is not mandatory. Instead, the government urges the public not to wear them in order to ensure sufficient supplies and personal protective equipment (PPE) for healthcare workers.
United States of America	Yes. On 2 April, the FDA approved antibody tests. Since then, they have been widely applied among the population. Commercially manufactured serologic tests that check for SARS-CoV-2 antibodies in individuals are becoming increasingly available for use through healthcare providers.	Despite its large population and density, it ranks sixth in performing the most RT-PCR tests, behind South Korea, Canada, Germany, and Italy.	Live information on the government’s web page. The Federal Communications Commission (FCC) supports and encourages citizen information.	$500 million for the Centre for Disease Control (CDC) to launch a new surveillance and data-collection system to monitor the spread of COVID-19.	Depending on the State, the beginning of the quarantine may fluctuate from 19March to 3 April.	Does not specifically advocate the use of surgical masks but does advise the use of “simple cloth face coverings” to slow the spread of the virus and prevent asymptomatic people from transmitting it to others. Mandatory use of masks depends on the state.
Venezuela	Yes. With limitations in acquiring rapid detection kits.	Yes; limited due to the excessive centralization of the RT-PCR sample processing in a single government laboratory. There is a mobile laboratory established on the border with Colombia. There is no periodical publication of epidemiological data.	Yes. Highly limited due to low diffusion and information control of government. The use of the WHO WhatsApp bot delivers real-time information to phones.	No	First country in Latin America to establish a lockdown (from 24 March to 27 April). Due to shortages of fuel and food, it was very difficult for the population to respect this order.	Mandatory use of masks. Random temperature check among citizens. China’s measures are being slowly replicated, according to Nicolas Maduro. (e.g., the use of “Carnet Patria”, which is similar to Quick Response (QR) codes but has a digital wallet where the government distributes economic bonds).
